# Early Physiological, Cytological and Antioxidative Responses of the Edible Halophyte *Chenopodium quinoa* Exposed to Salt Stress

**DOI:** 10.3390/antiox12051060

**Published:** 2023-05-07

**Authors:** Aymen Souid, Lorenza Bellani, Eliana Lanfranca Tassi, Karim Ben Hamed, Vincenzo Longo, Lucia Giorgetti

**Affiliations:** 1 Institute of Biology and Agricultural Biotechnology (IBBA), National Research Council, Pisa Unit, 56124 Pisa, Italy; aymen.souid@ibba.cnr.it (A.S.); vincenzo.longo@ibba.cnr.it (V.L.); 2 Laboratoire des Plantes Extrêmophiles, Centre de Biotechnologie de Borj Cedria, BP 901, Hammam Lif 2050, Tunisia; karimbenhamed2016@gmail.com; 3 Department of Life Sciences, University of Siena, 53100 Siena, Italy; 4 Research Institute on Terrestrial Ecosystems (IRET), National Research Council, 56124 Pisa, Italy; elianalanfranca.tassi@cnr.it

**Keywords:** antioxidant activity, antioxidant enzymes, *quinoa*, polyphenols, flavonoids, cytological analysis, mineral nutrient content, salt stress, seed germination, seedling growth

## Abstract

Quinoa (*Chenopodium quinoa* Willd.) is a plant of South American origin recently valorized for its nutritional and nutraceutical properties in human diet. Quinoa is cultivated in many parts of the world, with a selection of varieties with good adaptability to extreme climatic conditions and salt stress. The variety Red Faro, native to southern Chile but harvested in Tunisia, was considered for salt stress resistance, considering its seed germination and 10-day seedling growth at increasing doses of NaCl (0, 100, 200 and 300 mM). Seedlings were spectrophotometrically analyzed for antioxidant secondary metabolites (polyphenols, flavonoids, flavonols and anthocyanins), antioxidant capacity (ORAC, oxygen radical absorbance capacity, DPPH*, 2,2-diphenyl-1-pic-rylhydrazyl), antioxidant enzyme activity (superoxide dismutase (SOD), guaiacol peroxidase (GPX), ascorbate peroxidase (APX) and catalase (CAT)) and mineral nutrient content in root and shoot tissues. Cytogenetic analysis of root tip was performed to check for meristematic activity and the possible presence of chromosomal abnormalities induced by salt stress. The results indicated a general increase in antioxidant molecules and antioxidant enzymes NaCl dose-dependent, no effects on seed germination but negative effects on seedling growth, and little effect on root meristems mitotic activity. These results indicated that stress conditions can induce an increase in biologically active molecules that could be used for nutraceutical purposes.

## 1. Introduction

Quinoa (*Chenopodium quinoa* Willd.) is a dicotyledonous annual plant that belongs to the Chenopodiaceae family, native to the Andean highland region of Lake Titicaca in Perù and Bolivia, which was, for centuries, a basic food of ancient Andean populations [1,2]. Quinoa produces seeds with a high starch content and natural appearance similar to true cereals, but, in effect, it belongs to a group of seeds called pseudocereal, both because of their botanical classification and lack of gluten [3].

Being gluten free, quinoa, in particular ground to produce flour for pasta and pastry goods, can be a suitable food for people suffering from gluten intolerance and celiac disease [4]. Moreover, quinoa seeds contain high levels of bioactive molecules and functional components with nutraceutical effects that can improve human nutrition and health [3]. Quinoa cultivation increased in recent decades [5] thanks to the capacity of this plant to grow in the presence of different pedoclimatic conditions, surviving and completing its life cycle in harsh, saline environments [6].

Soil salinity is among the major abiotic stresses that plants must confront, mainly in arid and semiarid areas. The tolerance to high salinity is a crucial agronomic trait to sustain and to preserve food production [6]. Crop yield is mainly affected by climate stability, environmental conditions, agronomic factors and nutrient availability in the soil. Abiotic stress creates adverse effects on multiple aspects of plant morphology, biochemistry and physiology, which are directly connected with the growth and yield of plants [7]. 

Quinoa’s tolerance to abiotic stresses is one of the reasons why the FAO declared 2013 as the International Year of Quinoa and promoted it as one of the crops that may alleviate world hunger and poverty [8]. As most halophytes, this plant has morphological traits implicated in salt stress tolerance, such as stomatal density or the presence of epidermal bladder cells for the temporary storage of salt, which can be scattered by strong wind or external stimuli [9]. Moreover, quinoa presents various strategies to combine salinity perception and stress signaling with internal developmental responses [8], such as the activation of the antioxidant system to protect the cellular components from toxic ions [9]. 

Recently, a biogeographical approach opened up new perspectives for the adaptation and cultivation of quinoa outside its region of origin in order to investigate the possibility of exploiting marginal lands affected by salinity, drought and very high temperatures, such as the Saharan agrosystem of Algeria [10]. In this context, many studies were performed to investigate the tolerance of different quinoa genotypes to salinity in terms of agronomic performance (growth and yield) or morpho/physiological changes evidencing salt tolerance [11,12], but fewer studies have analyzed the effects of salinity on the nutritional quality of quinoa seeds.

Recently, an increase in total polyphenolics content (TPC) and antioxidant activity (AA) in quinoa plants grown under salinity was reported, suggesting a positive effect on the content of these important bioactive compounds [12]. Other results showed that salinity increased the concentration of bioactive molecules (TPC and AA) in a genotype-dependent manner [13]. 

The aim of the present research was to investigate the effects of NaCl treatments (100, 200 and 300 mM) on quinoa var. Red Faro seedlings by analyzing seed physiological response in terms of germination percentage, seedling growth, cytological analysis of root meristems, changes in the total polyphenols, total flavonoids, flavonols, anthocyanins, antioxidant activity (DPPH* and ORAC), mineral nutrient content and antioxidant enzymes. This approach might give new insights into the resistance to salt stress and consequent modifications in nutritional value of the under-analyzed variety grown in Tunisia. 

## 2. Materials and Methods

### 2.1. Seed Germination and Seedling Growth Conditions

*Chenopodium quinoa* Willd. seeds, Red Faro variety, native to southern Chile, were kindly provided by the Association of Environment and Development of Soliman (AEDS, Soliman, Tunisia). 

Seeds were sown in Petri dishes with two sheets of Whatman filter papers imbibed with distilled water for control and in the presence of NaCl 100, 200 and 300 mM for salt stress conditions. Seeds were germinated at 24 °C in the dark for 4 days, then exposed to daylight until the 10th day for the analysis of the earlier stages of germination and growth. Twenty seeds per dish, for a total of 100 seeds, were sown for each treatment. The germination percentage (G%) and the length of roots and shoots, as estimation of plant growth, were measured 10 days after sowing. 

### 2.2. Total Content of Polyphenols, Flavonoids, Flavonols and Anthocyanins

All the determinations were performed on seedlings that were carefully washed and separated in roots and shoots on the 10th day after sowing. For polyphenols, flavonoids and flavonols analyses extraction was carried out based on previous protocols [14]. Briefly, fresh material (1 g) was added to ethanol (80%, *v*/*v*) to reach a final concentration of 10 mg mL^−1^. The suspension was shaken overnight in the dark and then centrifuged at 3000× *g* for 20 min at 4 °C. The supernatant was recovered and processed or stored at –20 °C for the different determinations. 

The total polyphenols content (TPC) was determined following the protocol of Singleton and Rossi [15] with modifications. In particular, 100 μL of each quinoa extract were added to 3 mL of fivefold diluted in distilled water Folin–Ciocalteau (Sigma-Aldrich GMBH, Sternheim, Germany). After 6 min of incubation, 2 mL of 20% Na_2_CO_3_ were added; the solution was stirred and incubated for 1 h at room temperature (RT). The absorbance was determined spectrophotometrically at 760 nm against a blank using a UV/visible spectrophotometer apparatus (Perkin Elmer, Lambda 365, Waltham, MA, USA,). The TPC was estimated through the calibration curve of gallic acid and expressed as mg of gallic acid equivalents per gram of fresh weight (mg GAE g^−1^ FW) of each extract. 

The total flavonoids content (FC) was determined by the protocol of Heimler et al. [16]. Specifically, 200 μL of each quinoa extract was mixed with 60 μL of 5% NaNO_2_ solution and 800 μL of distilled water and incubated at RT for 5 min. Then, 60 μL of AlCl_3_ 10% in water was added and the solution incubated for 6 min. Next, 400 μL of 1 M NaOH and 480 μL of distilled water were added. After 5 min at RT the absorption was measured at 510 nm against a blank. The FC was determined through the calibration curve of quercetin and expressed as mg catechin equivalents (mg CE g^−1^ FW) of each extract.

The total flavonols content (FLC) was determined following the protocol of Romani et al. [17]. A total of 25 μL of each quinoa extract was added to 225 μL EtOH 10%, 1 mL HCl 2% and 250 μL HCl 0.1% in EtOH 95%, incubated for 30 min at RT and the absorption measured at 360 nm. The FLC was expressed as mg quercetin equivalents (mgQE g^−1^ FW) of extract. 

The total anthocyanin content was determined spectrophotometrically with the method of Landi et al. [18] with some modifications. Anthocyanins were extracted in acidified methanol (0.1% HCl, *v*/*v*) at room temperature, and their absorbance was measured at 535 nm. The absorbance values were converted into cyanidin-3-O-glucoside equivalents calculated on the basis of the molar extinction coefficient of this substance in the same solvent and expressed as mg cyanidin 3-glucoside eq/100 g FW.

### 2.3. Antioxidant Activity by Chemical Assays and Antioxidant Enzymes

Antioxidant activity by 2,2-diphenyl-1-picrylhydrazyl (DPPH*) assay was determined by the method of Boudjou et al. [19]. A solution of 60 μM DPPH* in MEtOH (1950 μL) was mixed with 50 μL of each quinoa extract, vortexed and incubated at 25 °C in the dark for 60 min. Absorbance at 517 nm was measured using MEtOH as a blank. As control, 50 μL of EtOH was used instead of extract. Antiradical activity (ARA) was expressed as percentage inhibition of the DPPH* radical by the following equation:

ARA = 100 × (1 − (absorbance of sample/absorbance of control))

The oxygen radical absorbance capacity (ORAC) assay was performed following the protocol of Ninfali et al. [20] with minor modifications. Briefly, 100 μL of each quinoa extract was diluted (1:10, 1:100:1:1000, *v*/*v*) and added to a mixture of 1 mL final volume. The mixture contained 800 μL sodium phosphate buffer (75 mM, pH 7.0) with fluorescein sodium salt (0.05 μM) plus a 100 μL solution of 2,2′-azobis(2-amidinopropane) dihydrochloride (400 mM). The standard mixture consisted of 100 μL of 50 μM 6-hydroxy-2,5,7,8-tetramethylchroman-2-carboxylic acid (Trolox); the control consisted of sodium phosphate buffer (75 mM, pH 7.0). The Perkin-Elmer Victor TM X3 apparatus (Waltham, MA, USA) measured fluorescence every 5 min at 37 °C at 485 nm excitation; 520 nm emission for 60 cycles. The ORAC values were calculated by the formula (As − Ab/At − Ab) × KA, where As is the area subtended by the curve (AUC) of fluorescein in the sample; At and Ab are Trolox and control AUCs, respectively. K is the dilution factor and A is the Trolox concentration (μM). The ORAC unit was expressed in micromoles of Trolox equivalents per g^−1^ FW (μmol g^−1^ TE).

For antioxidant enzyme determination, superoxide dismutase (SOD), guaiacol peroxidase (GPX), ascorbate peroxidase (APX), catalase (CAT) and total protein analysis extractions were carried out in accordance with Pereira et al. [21].

Quinoa roots and shoots were frozen and ground in liquid nitrogen, extracted in 100 mM potassium phosphate buffer (pH 7.5) added with 1 mM EDTA, 3 mM DTT and 5% (*w*/*v*) insoluble PVP in the ratio of 1:3 (*w*/*v*). The homogenate was filtered by cheesecloth, then centrifuged at 14,000× *g* for 30 min. The supernatant was collected and stored at −80 °C until use. Protein concentration in quinoa extracts was evaluated in accordance with the Bradford method [22], using bovine serum albumin (BSA) as a standard.

SOD activity was determined in accordance with Giannopolitis and Ries [23]. One unit of SOD activity was defined as the amount of protein inhibiting 50% of the initial reduction in NBT nitroblue tetrazolium under illumination, expressed as U mg^−1^ protein.

CAT activity was assayed in accordance with Aebi [24] considering changes in absorbance at 240 nm for the consumption of H_2_O_2_ and expressed as U mg^−1^ protein min^−1^. 

GPX activity was determined in accordance with Chance and Maehly [25] by absorbance change at 470 nm after H_2_O_2_-induced guaiacol oxidation, expressed as U mg^−1^ protein min^−1^. 

APX activity was evaluated in accordance with Nakano and Asada [26] by the decrease in absorbance at 290 nm following ascorbate oxidation (absorbance coefficient of 2.8 mM^−1^ cm^−1^).

### 2.4. Cytological Analysis of Root Meristems

Root meristems of quinoa seedlings from control and from each NaCl treatment were collected on the 3rd day after seed imbibition. Roots meristems were squashed on slides after fixation (ethanol/glacial acetic acid (3:1 *v*/*v*) overnight) and Feulgen staining [27]. Five root apices for each quinoa treatment were randomly analyzed by light microscope, counting at least 1000 nuclei per slide (for a total of 5000 nuclei). Feulgen-stained root meristems were also observed under a fluorescence microscope at 560 nm, wavelength specific for pararosaniline [28] to have a better resolution of small quinoa chromosomes.

Mitotic index (MI, number of mitosis per 100 nuclei) was determined for mitotic activity and possible cytotoxic effects on root meristems under treatments. Cytological anomalies (CA, number of mitotic anomalies per 100 mitoses), consistent in c metaphases, chromosomal bridges and fragments, lagging chromosomes and disturbed anaphases were analyzed for genotoxic effect estimation. 

### 2.5. Mineral Nutrient Content

At the harvesting, seedlings were separated in roots and shoots tissues, washed in deionized water, oven-dried until constant weight and weighed for the biomass determination. The dried roots and shoots were powdered (<1 mm) and the acid was digested, then they were analyzed for the content of macronutrients (Na^+^, Ca^++^, Mg^++^, K^+^) using an Inductively Coupled Plasma Emission Spectroscopy (ICP-OES, 5900 Agilent). 

A digestion method was performed following the protocol described in [29], with overnight pre-digestion in a mixture of HNO_3_/H_2_O_2_ (2.5:1, *v*/*v*) and microwave-assisted acidic digestion using a microwave Ethos 900 (FKV Srl, Bergamo, Italy). 

### 2.6. Statistical Analysis 

Analysis of variance (ANOVA) and a post hoc Tukey’s multiple range test was used to identify statistically significant differences between treatments using the Statistica package (StatSoft) 6.0 version. Different letters indicate significative differences at *p* ≤ 0.05. Data are the average of three separate experiments’ ± standard deviation (SD). Three separate experiments were conducted in triplicate and data were reported as mean ± SD. 

Pearson’s correlation and principle component analysis (PCA) were performed to visualize the response to salt stress both in quinoa roots and shoots.

## 3. Results and Discussion

### 3.1. Seed Germination and Seedling Growth 

**Figure 1:**
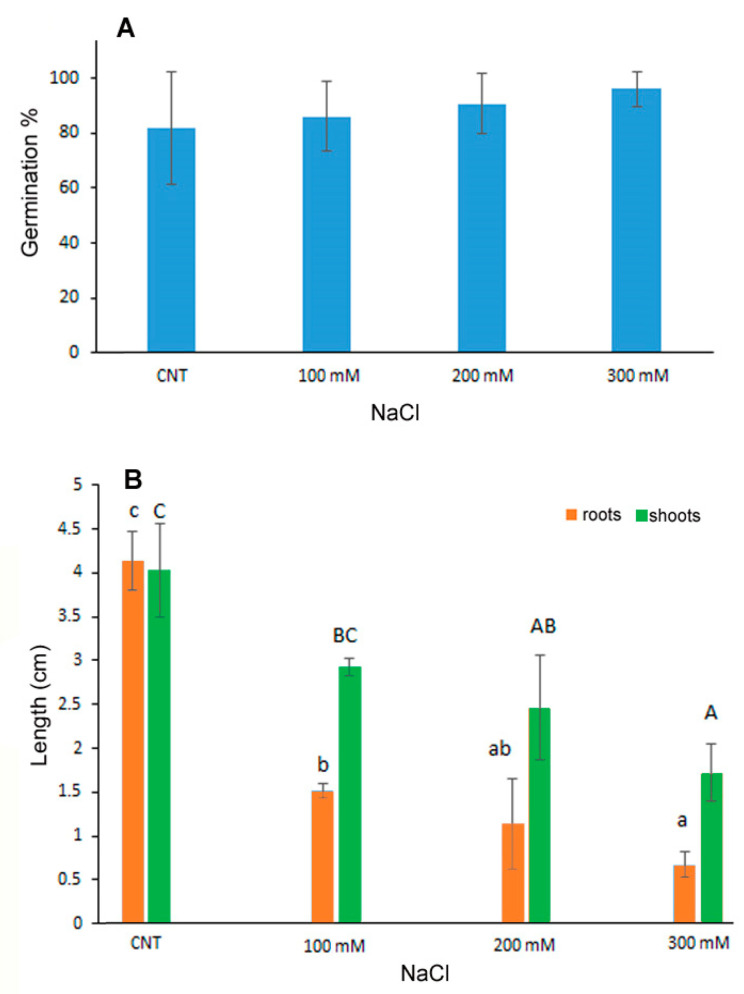
Germination percentage (**A**), root and shoot length (**B**) of 10-day control (CNT) and 100, 200 and 300 mM NaCl-treated seedlings of *Chenopodium quinoa*. Data are the average of three separate experiments’ ± SD. Different letters indicate significant differences among control and treatments according to Tukey test at *p* ≤ 0.05. Lowercase letters: roots; uppercase: shoots.

The percentage of germination of quinoa seeds exposed to saline stress with 100, 200 and 300 mM NaCl was not influenced by the treatments as no significant differences were observed with respect to the control seeds germinated in water (Figure 1A). On the contrary, the development of seedlings, in terms of shoot and root length, was inhibited in a dose-dependent manner at increasing NaCl concentrations (Figure 1B), thus indicating that despite germination the subsequent growth of the seedling was influenced by salt treatments. In particular, the root length was significantly reduced at 100 mM NaCl of about 1/3 with respect to control, with further reduction at increasing NaCl concentrations. Shoot length, instead, decreased significantly only at 200 and 300 mM NaCl of about 40 and 60%, respectively. 

The germination process can be inhibited by salt stress for the decrease in water potential and the accumulation of toxic Na^+^ and Cl^−^ ions mainly in glycophytic plants [6]. Moreover, it was demonstrated that quinoa is a facultative halophytic plant in which germination and seedling development can be influenced by particularly high salt concentrations; the resistance threshold depends on the genotype [6,30]. Quinoa var. Red Faro was previously investigated for salt resistance on 1-month-old plantlets starting NaCl treatments at the same salt concentrations used in the present experiment [31,32]. The analysis of photosynthetic efficiency in salt stress conditions evidenced functional and structural impairment of chloroplast performance at the highest concentration of 300 mM [31], but data on germination and seedling development were lacking [32]. 

On the contrary, data reported for other quinoa varieties (Titicaca, Puno and Vikinga) evidenced significant negative effects on the hypocotyl length already at 50 mM NaCl, with complete prevention of the growth at 300 mM for the Titicaca and Puno varieties [33]. 

### 3.2. Cytogenetic Analysis of Root Meristems

A previous cytogenetic study indicated that the quinoa genome is allotetraploid with a basic chromosome number x = 9 and 36 small size somatic chromosomes (2n = 36) [34,35]. In our analyses, quinoa small chromosomes were observed both by light microscope and by fluorescence microscope after Feulgen staining at 560 nm, wavelength specific for pararosaniline [28]. The latter gives a better resolution and a more accurate evaluation of mitotic index and chromosomal anomalies in salt stress conditions [27].

**Figure 2:**
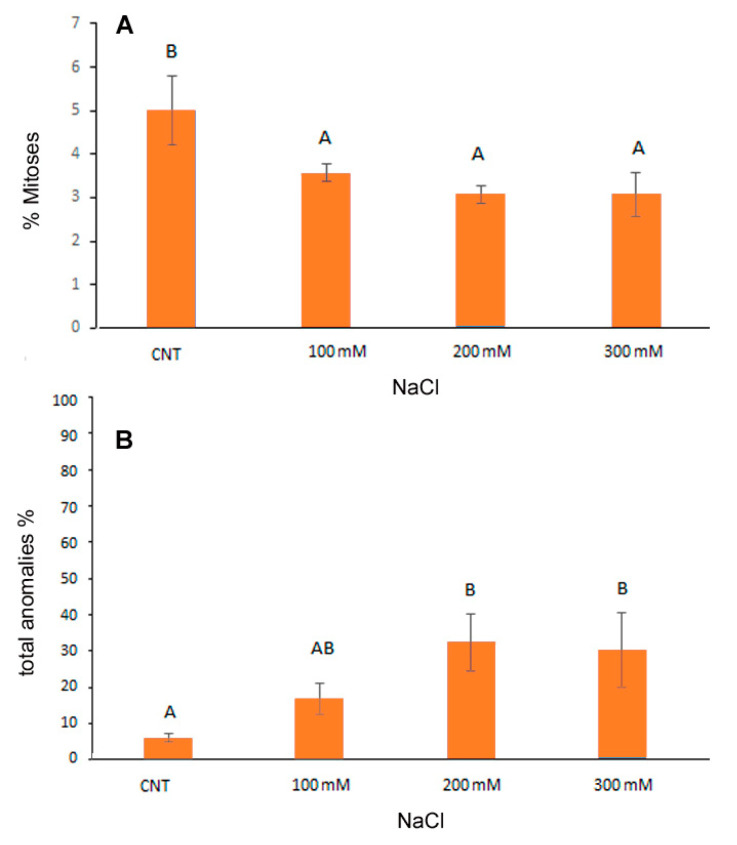
Cytological analysis of 5-day control (CNT) and 100, 200 and 300 mM NaCl-treated root meristems of *Chenopodiun quinoa* at light microscope after Feulgen staining. Mean values of mitotic index (**A**); % of total cytological anomalies (abnormal metaphases + abnormal ana/ telophases) (**B**). Data are the average of three separate experiments’ ± SD. Different letters indicate significant differences among control and treatments according to Tukey test at *p* ≤ 0.05.

Cytogenetic analysis showed a significant reduction in the mitotic index induced by NaCl from the lowest to the highest concentrations of NaCl in the range of −28.8% −40% with respect to control (Figure 2A). Considering the total percentage of abnormal mitotic phases (Figure 2B), both at 200 and 300 mM NaCl, the anomalies increased significantly, reaching about 30% of the total mitotic divisions observed.

The frequency (%) of the different mitotic phases was reported in Table 1. At the two highest salt concentrations a significant increase in abnormal metaphases (27.1% and 23.6% in 200 and 300 mM NaCl, respectively) in comparison to the control (4.1%) was observed. Additionally, abnormal telophases increased in salt treated root meristems (from 1.8% in control to 6.8% in 300 mM NaCl) but in this case differences were not statistically significant. The increase in the abnormal mitotic phases mainly influenced the frequency of the prophases which significantly decreased in the 300 mM treatment (from 24.1% of total mitoses in control and in 100 mM to 9% in 300 mM treated root meristems).

**Figure 3:**
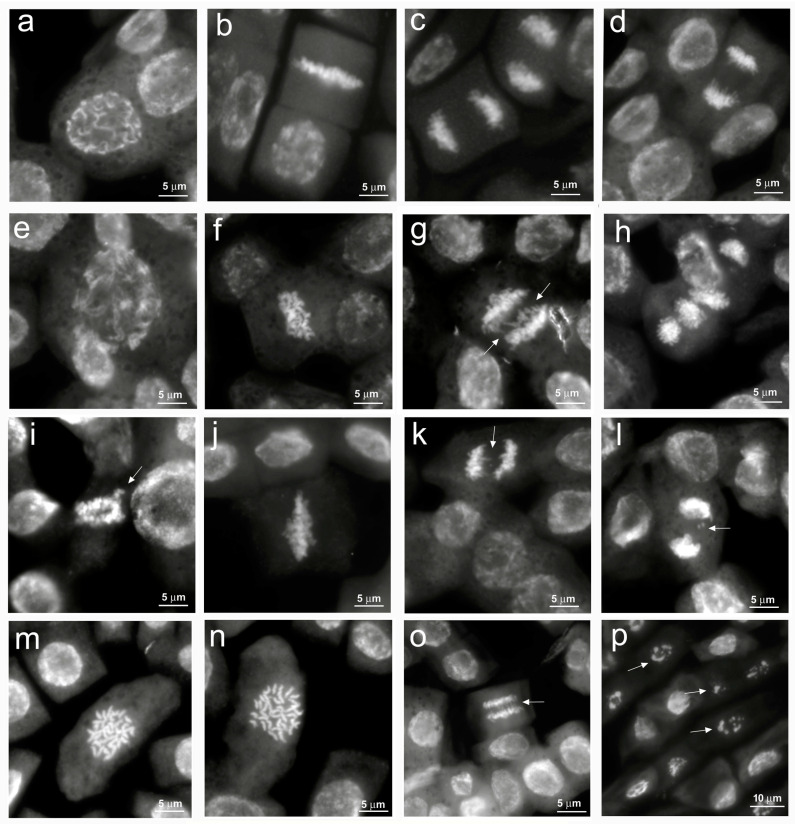
Cytological analysis of 5-day control and 100, 200, 300 mM NaCl-treated root meristems of *Chenopodiun quinoa.* (**a**–**d**) control: (**a**) Normal prophase; (**b**) Normal metaphase; (**c**,**d**) Normal anaphase; (**e**–**h**) 100 mM NaCl treatment: (**e**) Large prophase with paired chromosomes; (**f**), c–metaphase; (**g**) Anaphase with chromosome bridges (arrows); (**h**) Anaphases with abnormal spindles. (**i**–**l**) 200 mM NaCl treatment: (**i**) Abnormal pro-metaphase with lagging chromosomes (arrow); (**j**) c–metaphase; (**k**) Anaphase with chromosome bridges (arrow); (**l**) Anaphase with chromosome fragments (arrow). (**m**–**p**) 300 mM NaCl treatment: (**m**,**n**) Large c-metaphases; (**o**) Anaphase with chromosome bridges (arrow); (**p**) Pycnotic nuclei (arrows).

The main cytological aberrations observed in samples treated with NaCl are reported in Figure 3. Normal mitoses (Figure 3a–d) were mainly present in control root meristems. C-metaphases (Figure 3f,j,m,n), abnormal spindle formation (Figure 3e,h), chromosome bridges, fragments and lagging chromosomes at metaphases and anaphases (Figure 3g,i,k,l,o) were observed in salt-treated root meristems. Apoptotic nuclei, indicating damages to the meristematic tissue, were observed only at the highest NaCl concentration (Figure 3p). In our analysis, it was not possible to observe micronuclei due to the small size of the quinoa chromosomes.

Previous studies have demonstrated that salt stress can induce DNA fragmentation up to the induction of apoptotic phenomena with the consequent formation of chromosomal abnormalities [36]. Among cytogenetic anomalies, c-metaphases (colchicine-like metaphases) are generated by a failed formation of the mitotic spindle, which leads to the nondisjunction of sister chromatids with arrest of mitosis and possible formation of polyploid cells. Alterations of cellular homeostasis due to salt stress (ROS production and endonuclease activation) can lead to the breakage and loss of fragments or entire chromosomes, resulting in micronuclei formation till the generation of apoptotic phenomena. 

### 3.3. Phenolic Compounds and Antioxidant Activity

**Figure 4:**
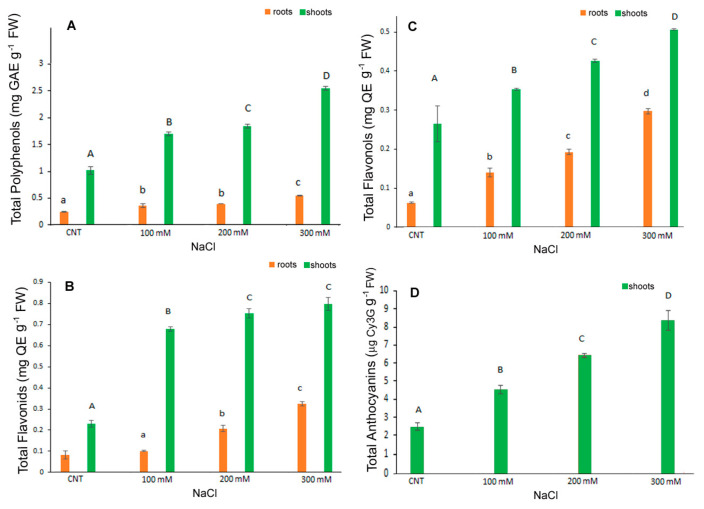
Contents of total polyphenols (**A**), total flavonoids (**B**), total flavonols (**C**), anthocyanins (**D**) in 10-day control (CNT) and 100, 200 and 300 mM NaCl-treated root and shoot of *Chenopodium quinoa* seedlings. Data are the average of three separate experiments ± SD. Different letters indicate significant differences among control and treatments according to Tukey test at *p* ≤ 0.05. Lowercase letters: roots; uppercase: shoots.

In the present research TPC, FC, FLC were determined separately in the shoots and roots of control and NaCl treated seedlings while total anthocyanins were determined in the entire seedlings. Results indicated a major content of TPC, FLC and FC in control shoots rather than in roots (more or less of five times) and a dose dependent significant increase in these metabolites under salt stress conditions in shoots. In particular, TPC was 0.24 mg GaE g^−1^ FW in control roots significantly increasing to 0.36, 0.39 and 0.55 mg GaE g^−1^ FW in 100, 200 and 300 mM NaCl treated roots, respectively (Figure 4A). In shoots TPC was 1.02 mg GaE g^−1^ FW in control and increased significantly to 1.71, 1.86, 2.57 mg GaE g^−1^ FW in 100, 200 and 300 mM NaCl treated shoots. 

FC and FLC values were in line with those of TPC, with the highest content in shoots and a gradual significant increase in roots and shoots at higher salt concentrations (Figure 4B,C). The FC values in roots were 0.08 mg CE g^−1^ FW in control and 0.1, 0.21 and 0.32 mg CE g^−1^ FW in 100, 200 and 300 mM NaCl-treated samples, respectively. The FC values in shoots were 0.23, 0.68, 0.75 and 0.79 mg CE g^−1^ FW in control, 100, 200 and 300 mM NaCl, respectively (Figure 4B). At the highest dose, therefore, shoots had a value of about four times higher than the control. 

The FLC (Figure 4C) values in roots were 0.06, 0.14, 0.19, 0.3 mg QE g^−1^ FW in control, 100, 200 and 300 mM NaCl, respectively. In shoots, the values were 0.26, 0.35, 0.42, 0.5 mg QE g^−1^ FW in control, 100, 200 and 300 mM NaCl, respectively, with a significant increase at each NaCl higher dose.

Anthocyanins content was determined in the shoots since in the roots it was not detectable (Figure 4D). Additionally, in this case, the salt stress induced a significant dose-dependent increase in anthocyanins being 2.47, 4.53, 6.44 and 8.36 µg cyanidin 3-glucoside eq g^−1^ FW in control, 100, 200 and 300 mM NaCl samples, respectively; the increase was more than three-fold at the highest dose.

**Figure 5:**
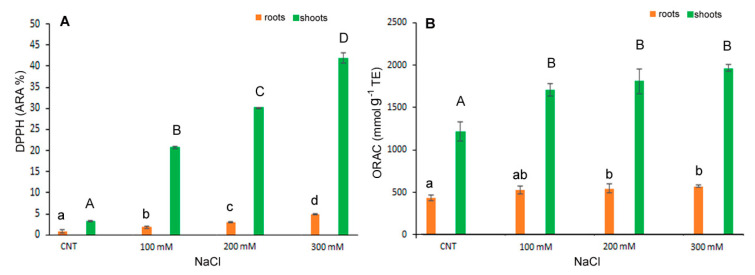
DPPH* scavenging activity (**A**) and ORAC (**B**) in 10-day control (CNT) and 100, 200 and 300 mM NaCl-treated root and shoot of *Chenopodium quinoa* seedlings. Data are mean ± S.E. of three replicates. Different letters indicate significant differences among control and treatments according to Tukey test at *p* ≤ 0.05. Lowercase letters: roots; uppercase: shoots.

A similar trend was observed for antioxidant capacity determined by DPPH* and ORAC assays (Figure 5A,B). The antiradical activity, measured as DPPH* quenching, gradually and significantly increased with salt stress either in shoot and root. In particular, in the shoot extracts, the DPPH* at 300 mM NaCl treatment was about 37 times higher than in control (Figure 5A). The ORAC assay revealed a significant increase at 100 mM NaCl and kept at similar level at the highest NaCl concentrations (Figure 5B). 

In our results, the increased antiradical activity and the high level of antioxidant molecules, polyphenols, flavonoids, flavonols and anthocyanin, particularly evident in the 300 mM NaCl treatment, might indicate their pivotal role in maintaining the balance between reactive oxygen species (ROS) generated by salt stress and the scavenging capacity of the antioxidant system in the detoxification of ROS in plants (18). In fact, recent works indicated that particularly flavonols and flavonoids, among polyphenols, were involved in plant protection being implicated in scavenging various forms of ROS [37]. Secondary metabolites, antioxidant activities and antioxidant enzymes can be influenced by biotic and abiotic factors. Their levels in quinoa can increase in response to salt stress or environmental conditions, as previously reported [13,38]. 

### 3.4. Antioxidant Enzyme Activities

**Figure 6:**
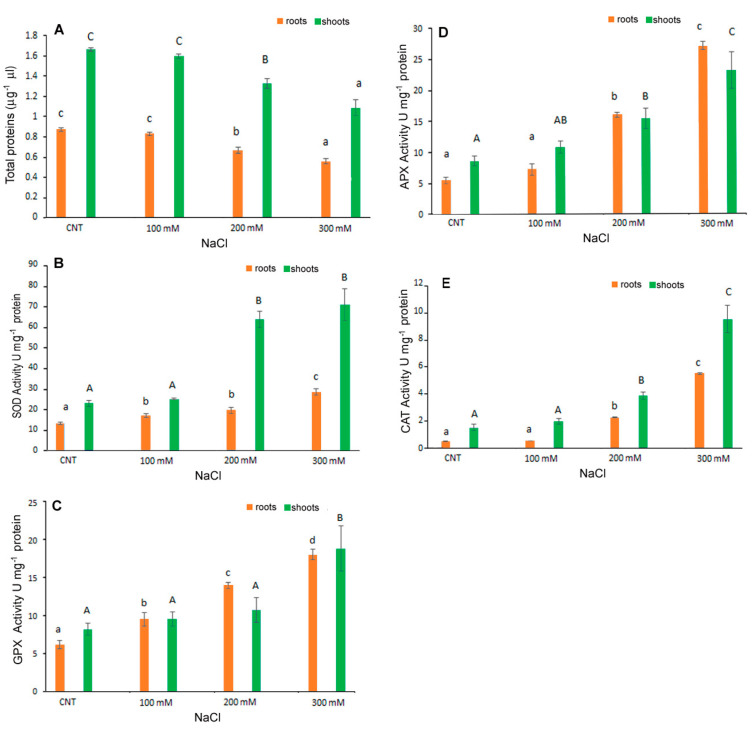
Total proteins content (**A**) and activity of SOD (**B**), GPX (**C**), APX (**D**) and CAT (**E**) in 10-day control (CNT) and 100, 200 and 300 mM NaCl-treated root and shoot of *Chenopodium quinoa* seedlings. Data are mean ± S.D. of three replicates. Different letters indicate significant differences among control and treatments according to Tukey test at *p* ≤ 0.05. Lowercase letters: roots; uppercase: shoots.

The level of antioxidant enzyme activities investigated in *C. quinoa* seedlings (Figure 6) showed a significant increase with the increase in NaCl concentrations. In shoots, APX and CAT activities were significantly higher with treatments of 200 and 300 mM NaCl than in control samples; SOD and GPX were significantly affected only by 100 mM NaCl. In particular, at 300 mM NaCl ~80 U mg^–1^ protein, 19 U mg^−1^ protein, 23 U mg^−1^ protein and 9.4 U mg^−1^ protein for SOD, GPX, APX and CAT, respectively, were detected in the shoots; ~28 U mg^–1^ protein, 18 U mg^−1^ protein, 27 U mg^−1^ protein and 5 U mg^−1^ protein for SOD, GPX, APX and CAT, respectively, were detected in the roots. 

The total protein content was also affected by salt stress with the lower content registered at 300 mM NaCl. Derbali et al. [32] reported that the antioxidant enzyme activities of two quinoa genotypes’ adult plants increased depending on the increase in NaCl concentrations. Causin et al. [30] investigated the putative antioxidant mechanism of enzymes involved in salt tolerance during germination and the early seedling growth of three quinoa genotypes. These authors indicated that a positive correlation for salt tolerance exists between seed germination and early seedling growth in some genotypes. The importance of the antioxidant enzyme metabolism in preventing oxidative damage under salt stress is confirmed by the present results for germinated quinoa var. Red Faro seeds.

### 3.5. Mineral Nutrient Content

**Figure 7:**
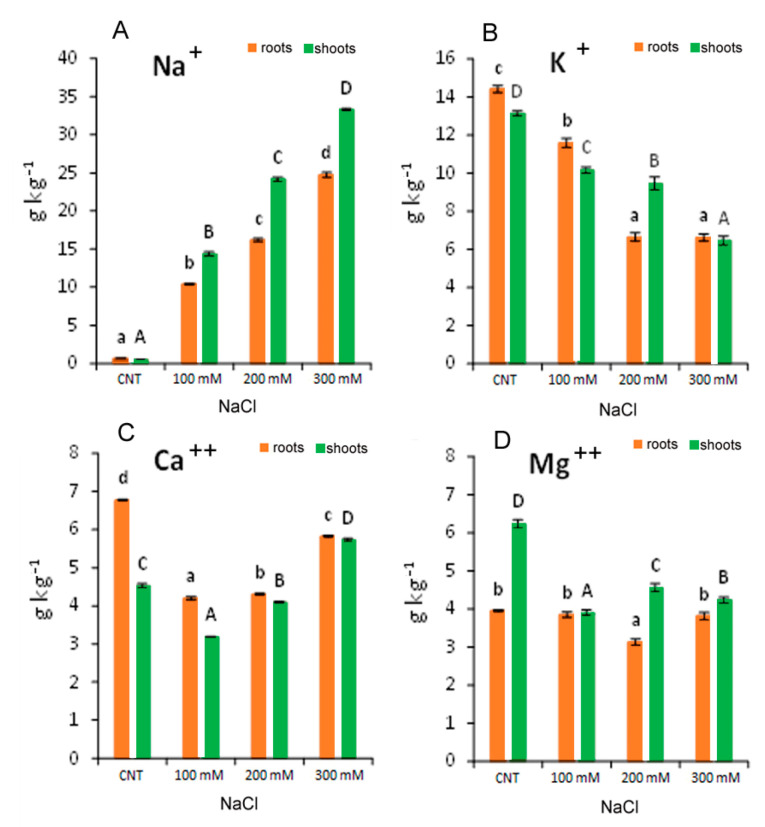
Concentration of elements: Na^+^ (**A**); K^+^ (**B**); Ca^++^ (**C**); Mg^++^ (**D**) in 10-day control (CNT) and 100, 200 and 300 mM NaCl-treated root and shoot of *Chenopodium quinoa* seedlings. Different letters indicate significant differences among control and treatments according to Tukey test at *p* ≤ 0.05. Lowercase letters: roots; uppercase: shoots.

Sodium and macronutrients composition in the roots and shoots of quinoa seedlings var. Red Faro were generally affected by the salt concentration in the growth media (Figure 7). Sodium concentration in the roots and shoots of all NaCl-treated plants increased drastically, being 15–28 times higher than the control plants (Figure 7A). A linear increase in Na in the roots and the shoots with the increasing salt concentration evidenced a correlation coefficient (R^2^) of 0.9915 and 0.9896, respectively. 

The macronutrients tested (K^+^, Ca^++^ and Mg^++^) were significantly affected by the salt stress. In particular, K^+^ in the roots and shoots decreased significantly with the increase in NaCl treatment, reaching a reduction of more than 50% at 300 mM with respect to the control (Figure 7B). In addition, at 100 mM NaCl, Ca^++^ was reduced by −30 and −38% in the roots and shoots, respectively, while at 300 mM NaCl, it was reduced by −14% in the roots and increased by +26% in the shoots, with respect to control (Figure 7C). Regarding Mg^++^, data evidenced that roots were less influenced by salt stress than shoots (Figure 7D). In the roots, Mg^++^ was reduced only at 200 mM NaCl (−20% with respect to the control), and in the shoots, it was reduced in all treatments from −27 to −37% with respect to the control (Figure 7D). Table 2 shows that the Na^+^/K^+^ ratio increased, both in the roots and in the shoots, with the increase in NaCl concentration in the growth media.

Our data suggested that the quinoa variety Red Faro has high capacity to uptake, translocate and accumulate high concentration of Na^+^ in the function of salinity in the growth media. It could be hypothesized that toxic Na^+^ (and other excess ions) were sequestered in the vacuoles or were subjected to a compartmentation mechanism, which will be responsible for the osmotic adjustment in plant cells/tissues, as already suggested for other quinoa varieties [32]. This excessive Na^+^ uptake led to a general nutritional imbalance. Sodium uptake was associated with the reduction in K^+^ both in the roots and in the shoots, which could be one factor influencing plant growth and development, as the regulation of osmotic pressure, the maintenance of cell turgor and the activation of enzymes employed in the metabolism and synthesis of proteins and carbohydrates are K^+^-dependent [39]. The reduced K^+^ content in quinoa was also a reason for the increased Na^+^/K^+^ ratio, which could reflect a competitive process with increased influx of Na^+^ at the expenses of K^+^ uptake. It has been suggested that Na^+^ could be used in osmoregulation to avoid its toxic effect, where several halophytes and the so-called glycophytic includers can perform an effective cellular portioning and translocate Na^+^ to the shoots to improve both crop resistance and Na^+^ content as a functional nutrient [40]. Moreover, the chemical and structural similarities between Na^+^ and K^+^ in hydrated forms could be partially causing the partial replacement of Na^+^ in some roles of K^+^ in plants [41]. In addition, the involvement of membrane transporters that catalyse Na^+^ movement have also been considered, such as the involvement of HKT-type transporters (High-affinity Potassium Transporters) for the translocation of Na^+^ from the root to the shoot via its recall from the xylem [41,42]. 

The decrease detected for Ca^++^ in the roots could be explained as a possible exchange of Na^+^ with Ca^2+^ from membranes and cell wall as a primary response to salt stress [43,44]. The pronounced effect observed at 100 and 200 mM NaCl suggested ion competition and reduced activity of Ca^2+^. The less marked decrease observed at 300 mM NaCl could indicate a tentative maintenance of Ca^2+^ level or its translocation to shoot under the increase in ionic strength in the solution with high salinity. In fact, the level of Ca^++^ in the shoot at 300 mM NaCl is higher than in control and in 100 and 200 mM NaCl-treated seedlings. The Mg^++^ concentration remained almost constant in the roots at any treatment, suggesting a lower replacement of Na^+^ in its functions, while a decrease was observed in the shoots. Thus, the reduction in the macronutrient’s uptake observed in quinoa var. Red Faro under salinity stress may be correlated with the reduced growth observed, although NaCl in solution modulated nutrient levels in roots and shoots differently. 

### 3.6. Pearson’s Correlation and PCA Analysis

**Figure 8:**
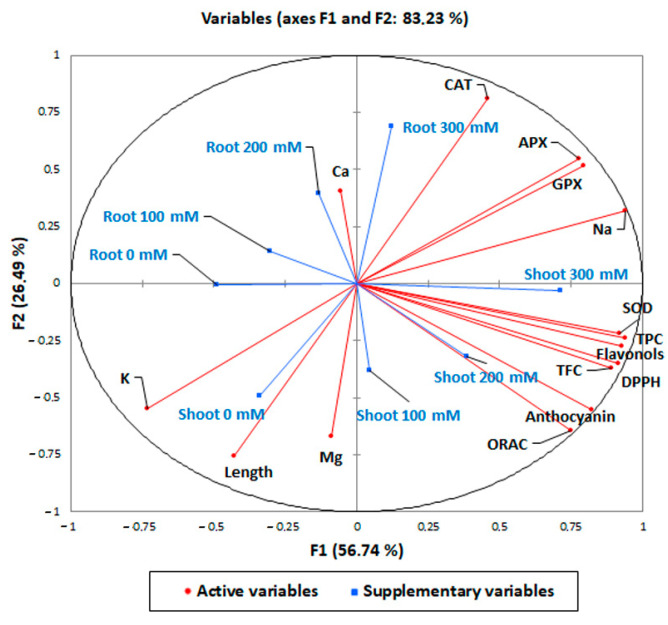
Principal component analysis of the shoot and root elongation, the content of total polyphenols, flavonols, total flavonoids, anthocyanins, DPPH* and ORAC scavenging activity, antioxidant enzymes (APX, GPX, SOD, and CAT) and mineral elements in 10-day control (0 mM) and 100, 200, 300 mM NaCl-treated root and shoot of *Chenopodium quinoa* seedlings.

In order to highlight the antioxidant and biochemical responses of quinoa var. Red Faro roots and shoots to increasing concentrations of NaCl, a Pearson’s correlation (Table 3) and a principal component analysis (PCA) were carried out (Figure 8). Our analysis identified several statistically significant correlations (positive or negative) between the tissues analyzed and the different parameters measured (Table 3). The strongest significant response to treatments was reported for root 300 mM NaCl-treated samples, which were significantly positively correlated with the antioxidant enzymes (CAT, r = 0.829; APX, r = 0.454; GPX, r = 0.588) and Na^+^ content (r = 0.319). Shoot 300 mM had the highest significant and positive correlation with antioxidant compounds (TPC, r = 0.776; TFC, r = 0.542, flavonols, r = 0.615; anthocyanins, r = 0.678), DPPH* (r = 0.738) and ORAC activity (r = 0.533) and antioxidant enzymes (SOD, r = 0.698; GPX, r = 0.523; APX, r = 0.657), thus confirming the highest antioxidant response to NaCl treatments. It is noteworthy that only root 300 mM NaCl-treated samples strongly negatively correlated with length (r = −0.500), while the correlation of shoot 300 mM NaCl did not reach significance (r = −0.182), in accord with results reported in Figure 1. The two components (PCs) contributed 83.23% to the cumulative variance, the PC1 (F1 axis) accounted for 56.74% of the existing variability and the PC2 (F2 axis) for 26.49% of the total variance (Figure 8). A full visualization of the distribution of variables across the first two PC spaces (Figure 8) confirmed the pattern of the pairwise correlations reported in Table 3 and described above.

## 4. Conclusions

The present results on quinoa seedlings evidenced that the var. Red Faro is tolerant to salt stress up to 300 mM NaCl, showing that the percentage of germination and the mitotic activity of root meristems were slightly affected by salt treatments, although an increase in the chromosomal aberrations was noticed, maybe induced by ROS production. In addition, salt treatments affected the mineral nutrient content of the seedlings and sodium accumulation in the tissues, which in turn can act as a chemical eustressor, Na^+^ being translocated to the shoots as a functional nutrient and improving health-promoting molecules, such as antioxidants and bioactive compounds. In fact, antioxidant enzymes and bioactive molecules increased in NaCl treatments in a dose-dependent manner. Interestingly, the significant increase in antioxidant molecules, such as polyphenols, flavonoids and flavonols, which have nutraceutical properties, make the plant, in particular this variety, a good candidate as a functional food for animal and human nutrition.

This study can be the basis for evaluation of the best doses and times of exposure in salt stress experiments in order to have a good compromise between plant growth, its content of mineral elements and the production of biologically active molecules of nutraceutical interest with antioxidant activity and lower anti-nutritional compounds in comparison to seeds. For their nutritional properties, 10-day quinoa seedlings, which are sprouts and microgreens, can be considered superfoods in the food industry and used either fresh or in commercial powder as dietary supplements for human wellness. 

## Figures and Tables

**Table 1:** Cytological analysis of Quinoa root meristems in control and after 72 h treatments with NaCl 100 mM, 200 mM and 300 mM. Normal and abnormal (Abn) prophases, metaphases and ana/telophases were expressed as mean values ± standard deviation on 100 mitoses analyzed.

	Control	NaCl 100 mM	NaCl 200 mM	NaCl 300 mM
%Prophases	24.1 ± 8.0 b	24.2 ± 5.8 b	23.8 ± 3.7 b	9.0 ± 3.1 a
%Metaphases	34.1 ± 5.5 b	15.1 ± 4.9 a	30.6 ± 8.3 b	34.3 ± 4.2 b
%Abn Metaphases	4.1 ± 0.7 a	11.0 ± 5 ab	27.1 ± 6.2 b	23.6 ± 9.7 b
%Ana/Telophases	35.9 ± 3.6 bc	44.0 ± 5.3 c	13.0 ± 3.3 a	26.2 ± 9.6 ab
%Abn Ana/Telophases	1.8 ± 1.7 a	5.7 ± 3 a	5.4 ± 1.9 a	6.8 ± 3.69 a

Values are means of at least three replicates ± SD. Different letters within rows denote significant differences at *p* < 0.05.

**Table 2:** Ratio of ions Na^+^/K^+^ under the NaCl treatments in roots and shoots.

NaCl (mM)	Na^+^/K^+^	
	Roots	Shoots
0	0.04	0.05
100	1.41	0.90
200	2.56	2.44
300	5.15	3.74

**Table 3:** Correlation coefficient of shoot and root elongation, the content of total polyphenols, flavonols, total flavonoids, anthocyanins, DPPH* and ORAC scavenging activity, antioxidant enzymes (APX, GPX, SOD, CAT) and mineral elements in 10-day control (0 mM) and 100, 200, 300 mM NaCl-treated root and shoot of *Chenopodium quinoa* seedlings. Values in bold are different from 0 with a significance level alpha = 0.05.

Variables	Length	TPC	TFC	Flavonols	Anthocyanin	DPPH	ORAC	SOD	GPX	APX	CAT	Ca	Na	Mg	K	Root 0 mM	Root 100 mM	Root 200 mM	Root 300 mM	Shoot 0 mM	Shoot 100 mM	Shoot 200 mM	Shoot 300 mM
**Length**	**1**	−0.185	−0.114	−0.225	0.090	−0.102	0.157	−0.183	**−0.642**	**−0.663**	**−0.730**	0.085	**−0.685**	**0.583**	**0.824**	**0.550**	−0.243	−0.359	**−0.500**	**0.515**	0.179	0.041	−0.182
**TPC**		**1**	**0.906**	**0.880**	**0.929**	**0.986**	**0.834**	**0.956**	**0.609**	**0.622**	0.196	−0.031	**0.802**	0.008	**−0.495**	−0.309	−0.254	−0.238	−0.165	−0.309	0.054	**0.445**	**0.776**
**TFC**			**1**	**0.938**	**0.927**	**0.948**	**0.925**	**0.846**	**0.513**	**0.431**	0.094	−0.291	**0.731**	0.066	**−0.474**	**−0.425**	−0.400	−0.257	−0.097	−0.227	0.382	**0.482**	**0.542**
**Flavonols**				**1**	**0.892**	**0.899**	**0.891**	**0.876**	**0.604**	**0.554**	0.256	−0.241	**0.782**	0.203	**−0.579**	**−0.593**	−0.381	−0.238	0.046	−0.041	0.2	0.394	**0.615**
**Anthocyanin**					**1**	**0.966**	**0.966**	**0.889**	0.362	0.357	−0.079	−0.195	**0.590**	0.283	−0.280	−0.328	−0.328	−0.328	−0.328	−0.030	0.217	**0.447**	**0.678**
**DPPH**						**1**	**0.900**	**0.922**	**0.530**	**0.524**	0.096	−0.120	**0.747**	0.055	**−0.442**	−0.318	−0.295	−0.266	−0.219	−0.261	0.191	**0.431**	**0.738**
**ORAC**							**1**	**0.789**	0.257	0.212	−0.176	−0.361	**0.496**	0.355	−0.222	**−0.408**	−0.35	−0.340	−0.323	0.075	0.374	**0.438**	**0.533**
**SOD**								**1**	**0.634**	**0.654**	0.258	0.011	**0.787**	0.145	**−0.486**	−0.352	−0.285	−0.237	−0.077	−0.176	−0.137	**0.566**	**0.698**
**GPX**									**1**	**0.932**	**0.839**	0.291	**0.893**	−0.327	**−0.787**	−0.312	−0.201	−0.097	**0.588**	**−0.482**	−0.201	0.182	**0.523**
**APX**										**1**	**0.829**	0.367	**0.879**	−0.336	**−0.811**	−0.287	−0.175	0.062	**0.454**	**−0.444**	−0.357	0.091	**0.657**
**CAT**											**1**	**0.453**	**0.665**	−0.37	**−0.743**	−0.222	−0.164	0.088	**0.829**	−0.357	−0.353	−0.122	0.301
**Ca**												**1**	−0.009	−0.058	0.078	**0.672**	−0.218	−0.181	0.342	−0.103	**−0.568**	−0.253	0.310
**Na**													**1**	−0.340	**−0.878**	**−0.516**	−0.178	0.023	0.319	**−0.522**	−0.041	0.299	**0.616**
**Mg**														**1**	**0.466**	−0.115	−0.158	**−0.475**	−0.175	**0.895**	−0.135	0.152	0.011
**K**															**1**	**0.6**	0.232	**−0.412**	**−0.417**	**0.433**	0.046	−0.046	**−0.436**
	−1	−0.9	−0.8	−0.7	−0.6	−0.5	−0.4	−0.3	−0.2	−0.1	0	0.1	0.2	0.3	0.4	0.5	0.6	0.7	0.8	0.9	1		

Values in bold are different from 0 with a significance level alpha = 0.05.

## Data Availability

The data are contained within the article.
